# A child with Chronic Nonbacterial Osteomyelitis and celiac disease: accidental association or two different aspects of the same condition?

**DOI:** 10.1186/s13052-025-01842-x

**Published:** 2025-01-30

**Authors:** Grazia Bossi, Maria Sole Prevedoni Gorone, Luca Lungarotti, Francesco Pelillo, Amelia Mascolo, Matteo Naso, Daniele Veraldi, Francesca Olivero, Costanza Chirico, Maria Vittoria Marino, Cristina Dutto, Gian Luigi Marseglia

**Affiliations:** 1 Department of Pediatrics, IRCCS Policlinico San Matteo Foundation, Viale Golgi 19, Pavia, 27100 Italy; 2 Department of Diagnostic and Interventional Radiology and Neuroradiology, IRCCS Policlinico San Matteo Foundation, Pavia, 27100 Italy; 3 Orthopedics and Traumatology Clinic, IRCCS Policlinico San Matteo Foundation, Pavia, 27100 Italy; 4 Pediatric School of Specialization, University of Pavia, Pavia, 27100 Italy; 5 Department of Clinical-Surgical Diagnostic and Pediatric Sciences, University of Pavia, Pavia, 27100 Italy

**Keywords:** Pediatrics, Chronic nonbacterial osteomyelitis, Autoimmune intestinal diseases, Bone inflammation

## Abstract

**Background:**

Chronic Nonbacterial Osteomyelitis (CNO) is a rare auto-inflammatory disease that mainly affects children, and manifests with single or multiple painful bone lesions. Due to the lack of specific laboratory markers, CNO diagnosis is a matter of exclusion from different conditions, first and foremost bacterial osteomyelitis and malignancies. Whole Body Magnetic Resonance (WBMR) and bone biopsy are the gold standard for the diagnosis. Although the association with Inflammatory Bowel Disease (IBD) has been reported in the literature, cases of CNO in celiac patients have never been described before.

**Case presentation:**

We report about a girl of 3 years and 8 months of age who presented with severe bone pain, slight increase of inflammatory markers, micro-hematuria and high calprotectin values. Her personal medical history was uneventful, apart from low weight growth. She had never complained of abdominal pain or other gastro-intestinal symptoms. WBMR showed the classical features of multifocal CNO, and biopsy confirmed the diagnosis. Celiac disease (CD) was suspected on the basis of antibody screening, and confirmed by gut biopsy. With gluten-free diet the patient achieved rapid and complete symptom remission together with healing of all the bone lesions proven by WBMR. Three years after the onset of the disease the girl is healthy and totally asymptomatic, still on clinical and radiological follow-up.

**Conclusions:**

Based on our experience, the diagnostic work-up of new cases of CNO should include the screening test for CD and, according to the literature, the possibility of IBD should also be properly ruled out. When CNO and CD coexist, gluten-free diet, combined with antinflammatory therapy, could be able to completely reverse bone lesions, shortening the duration of medical treatment. Because the diseases’ onset is seldom simultaneous, patients with CNO and IBD deserve a properly extended follow-up. Finally, the analysis of the relationship between CNO and autoimmune intestinal diseases provides a unique opportunity to understand the pathophysiological pro-inflammatory network underlying both types of disorders and it is necessary to make the most suitable therapeutic choice.

## Background

Chronic Non-Bacterial Osteomyelitis (CNO) and Chronic Recurrent Multifocal Osteomyelitis (CRMO) are terms often used interchangeably to define a rare auto-inflammatory bone disorder, mostly affecting children and adolescents and rarely seen in adults, known for over fifty years [1].

Over the years, it has become evident that the term CRMO only refers to the most severe form of the disease, characterized by recurrent multifocal bone lesions [2], while the term CNO best fits the clinical heterogeneity of this multifaceted disorder, which includes a wide range of clinical findings, from single to multiple bone lesions, often symmetric, sometimes relapsing [3], seldom associated with skin involvement (synovitis, acne, pustolosis, hyperostosis and osteitis syndrome, SAPHO) [4].

Although CNO is still regarded as a rare disease, with an estimated incidence ranging between 0.4 and 2 per 100.000 children per year, its actual prevalence has been overlooked due to problems of missed diagnosis and underreporting [5]. The recent increase in cases reported by some referral centers is probably only the effect of an improved awareness of the disease [6, 7].

Most pediatric cases are reported between 8 and 13 years, with a median age at diagnosis of 10 years. Disease onset before 3 years and after 18 years of age is rare [3].

Ethnicity and environment are not considered contributing factors, while female sex definitely seems to be a risk factor, at least in European cohorts, where the affected females are twice the males [8–12].

Clinical features of CNO are unspecific and highly variable, mostly depending on the number and position of the bone lesions. The onset is usually insidious and the disease may run a chronic or a recurrent course. Common symptoms at disease onset include bone pain with or without tenderness and swelling. Some patients experience low-grade fever, malaise, fatigue, night sweats and weight loss [3, 13]. Any segment of the skeleton can be affected, but long bones metaphysis, pelvis, clavicle, mandible and spine are the main sites of involvement; skull is almost never affected. Localization at sternum, clavicle and jaw is very suggestive of CNO [14]. Spinal involvement, evident in about a third of cases, must be actively sought even in asymptomatic cases because it can result in compressive vertebral fracture. Bone lesions are mostly multiple and asymmetric, but usually clavicle involvement is unifocal and mainly localized in the proximal third [15–18].

Laboratory findings are not helpful for the diagnosis, because the traditional inflammatory markers (C-reactive protein, CRP; erythrocyte sedimentation rate, ESR; ferritin, leukocytosis) can be normal or at least only slightly increased during the disease flares and return to normal ranges during inactive periods. Anyway, they are never predictive of the disease extent or of its course [19–21].

The CNO diagnosis relies on radiological assessment and, among the different tools available, whole-body magnetic resonance imaging (WBMR) remains the gold standard. Compared to classic X-ray, computed tomography, and scintigraphy, WBMR offers a more sensitive method to detect silent lesions, especially in the early stage of the disease, and complications, as vertebra plana and fractures. Moreover, WBMR spares children from radiation and is therefore the best method for disease monitoring [22].

Due to its rareness and unspecific clinical presentation, CNO diagnosis is often delayed, with a mean interval of 12 months between symptom onset and diagnosis [3, 12, 20]. The diagnostic delay can be as long as five years [23], and seems to be associated with a worse outcome [12, 24]. The typical MR findings vary according to the progression of the inflammatory bone lesions. While bone marrow edema is the hallmark of the early stage of the disease, osteolysis and/or sclerosis with cortical thickening are commonly seen in the later stages [14, 22].

That of CNO is a diagnosis of exclusion and bone biopsy still remains a cornerstone, mainly to exclude other causes. The differential diagnosis includes acute septic osteomyelitis above all, but also primary bone malignancies (osteosarcoma, Ewing sarcoma, rhabdomyosarcoma, leukemia and lymphoma) and benign tumors (osteoid osteoma), trauma, metabolic disorders (hypofosphatasia), Langerhans Cell Histiocytosis, different auto-inflammatory disorders (DIRA, PAPA), osteonecrosis [25, 26]. Bone biopsy typically shows features of inflammation in the absence of infection [10], and histologic features depend on disease progression, with predominance of innate immune cells (neutrophils and monocyte/macrophages) in early stages and during the disease flares, while adaptive infiltrates (lymphocytes and plasma cells) can be detected in later course or during remission between phases of disease activity [9].

The etio-pathology of CNO is still unknown, but an increasing number of scientific evidence suggests a multifactorial mechanism, with the interplay of genetic predisposition, cytokine dysregulation, and osteoclastic activation acting in synergy to induce sterile bone lesions. The pivotal factor in CMO pathogenesis seems to be related to an aberrant regulation in the immune system, resulting in a cytokine/chemokines dysregulation. The imbalance between the overproduction of pro-inflammatory cytokines (IL-1β, IL-6, TNF-α, IL-20) and the underproduction of immuno-regulating cytokines (IL-9, IL-10, IL-19), shown by the monocytes of CNO patients, seems to be responsible for bone inflammation. Moreover, the increased expression of NLRP3 inflammosome and of IL-1β mRNA demonstrated in the peripheral blood mononuclear cells from CNO patients could be responsible for the accelerated osteoclast differentiation and activation, ultimately leading to inflammatory bone loss [27, 28].

With regard to genetic predisposition, despite the growing number of candidate genes reported in the literature, many of these reports are only anectodal cases [8, 29–33] or findings in animal models [34] not reproduced in humans [35] or later denied [36]. Moreover, how the genetic anomalies can lead to bone damage still remains unclear [28].

Since neither guidelines, protocols, nor expert consensus for treatment are available, CNO therapy still remains essentially empiric, mainly focused on inflammation control, pain relief and prevention of bone complications. Non-steroidal anti-inflammatory drugs (NSAIDs), naproxen in particular, are considered the first-line choice in pediatric patients without spine involvement. In several case series they proved to be able to induce significant clinical improvement and bone lesions decrease in a percentage of patients varying between 43 and 83% [8, 19, 25]. Second-line therapy for patients who do not respond to NSAIDs or with spinal CNO implies the use of short course of steroids, non-biological disease modifying agents (methotrexate, sulfasalazine) [20, 37], TNF-alfa inhibitors (infliximab, etanercept) [38], bisphosphonates (pamidronate, alendronate) [39, 40], and IL-1 inhibitors (anakinra) [41]. Among all the proposed treatment for NSAIDs-refractory or spinal CNO, bisphosphonate seems to be the most useful option, especially in patients with multifocal disease [40].

CNO is a chronic disease, mostly self-limiting, often with a waxing and waning character and alternate phases of flares and remission that may continue into adulthood. If treated early, its prognosis is good, with overall remission rates ranging from 50 to 80% [16]. Apart from spinal localization, male sex and multifocal involvement at disease onset are considered poor prognostic factors [3]. Moreover, the favorable outcome may be hampered by some severe complications, such as scoliosis, kyphosis, decrease joints function, asymmetric limb length [6].

In up to 30% of patients CNO is associated with other conditions, mostly inflammatory disorders, involving different organs or systems, such as skin (severe acne, psoriasis, palmoplantar pustolosis, Sweet syndrome, pyoderma gangrenosum, erythema nodosum), blood vessels (Takayasu arteritis); joints (Juvenile Idiopatic Arthritis; spondyloarthritis) [3, 12], kidney [42], liver (sclerosing cholangitis), and bowel [42–44]. The bowel autoimmune disorder most often reported in association with CNO is Inflammatory Bowel Diseases (IBD), with Crohn’s disease being more frequent than Ulcerative Colitis [44, 45].

Surprisingly, despite CD being one of the most frequent pediatric gastrointestinal disease all over the world, the association between CNO and CD has never been described.

We report on diagnosis and long-term follow-up of a child simultaneously diagnosed affected by CNO and CD, who achieved prompt and complete clinical and radiological remission of multiple bone lesions with gluten-free diet combined with a very short course of antinflammatory therapy.

## Case presentation

A 3 years and 8 month old Caucasian girl, only child of unrelated healthy parents, came to our attention for slight left knee swelling, lower limbs pain and increasing walking difficulties lasting one week. She had been regularly vaccinated according to the current national schedule and her past medical history was only remarkable for psychomotor development delay and poor weight gain, both never investigated; she had never complained of abdominal pain, constipation, diarrhea or vomiting. The referred symptoms had manifested shortly after a mild and self-resolving episode of upper respiratory infection and had progressively worsened up to the total refuse of standing and walking despite the ongoing symptomatic treatment therapy (intermittent ibuprofen). No fever was reported. Blood tests performed a few days before had showed only a mild increase of the ERS value (30 mm/h, nv. < 15), while the left knee ultrasonography revealed a minimum and non-quantifiable intra-articular effusion.

In the emergency room the girl presented pale and suffering but afebrile, with low weight (−2 SD according to the WHO Child Growth Standard), very poor speech and total refusal of relationship with strangers. The physical examination showed severe limitation of active and passive mobilization of lower limbs and a mild tenderness of the left knee. Neurological evaluation was normal, in particular deep tendon reflex could be normally elicited.

The girl was then admitted to our paediatric department to continue the diagnostic work-up; in the meanwhile the empiric anti-inflammatory therapy was continued intravenously (ibuprofen, 30 mg/kg/day in 3 doses) although with limited benefit. Apart from a slight increase in ERS value (41 mm/h) and platelet count (608.000/mmc^3^), all other laboratory tests, including LDH, uric acid, alkaline phosphatase, C3 and C4 complement fraction, were within the normal range. Because the urinalysis showed persistent, isolated, mild microscopic haematuria and the calprotectin value was slightly elevated (365 ng/ml), in absence of macroscopic or microscopic blood, we performed an abdomen ultrasound that showed a not significant dilatation of right renal pelvis and a normal thickness of the intestinal walls. All the serological tests and the cultural examination performed on different biological materials (blood, urine, stool, nasal and throat swabs) failed to detect an infective agent. The ultrasound conformed the minimal joint effusion in the left knee, while X-rays of hips, knees and vertebral column were completely normal.

**Fig. 1:**
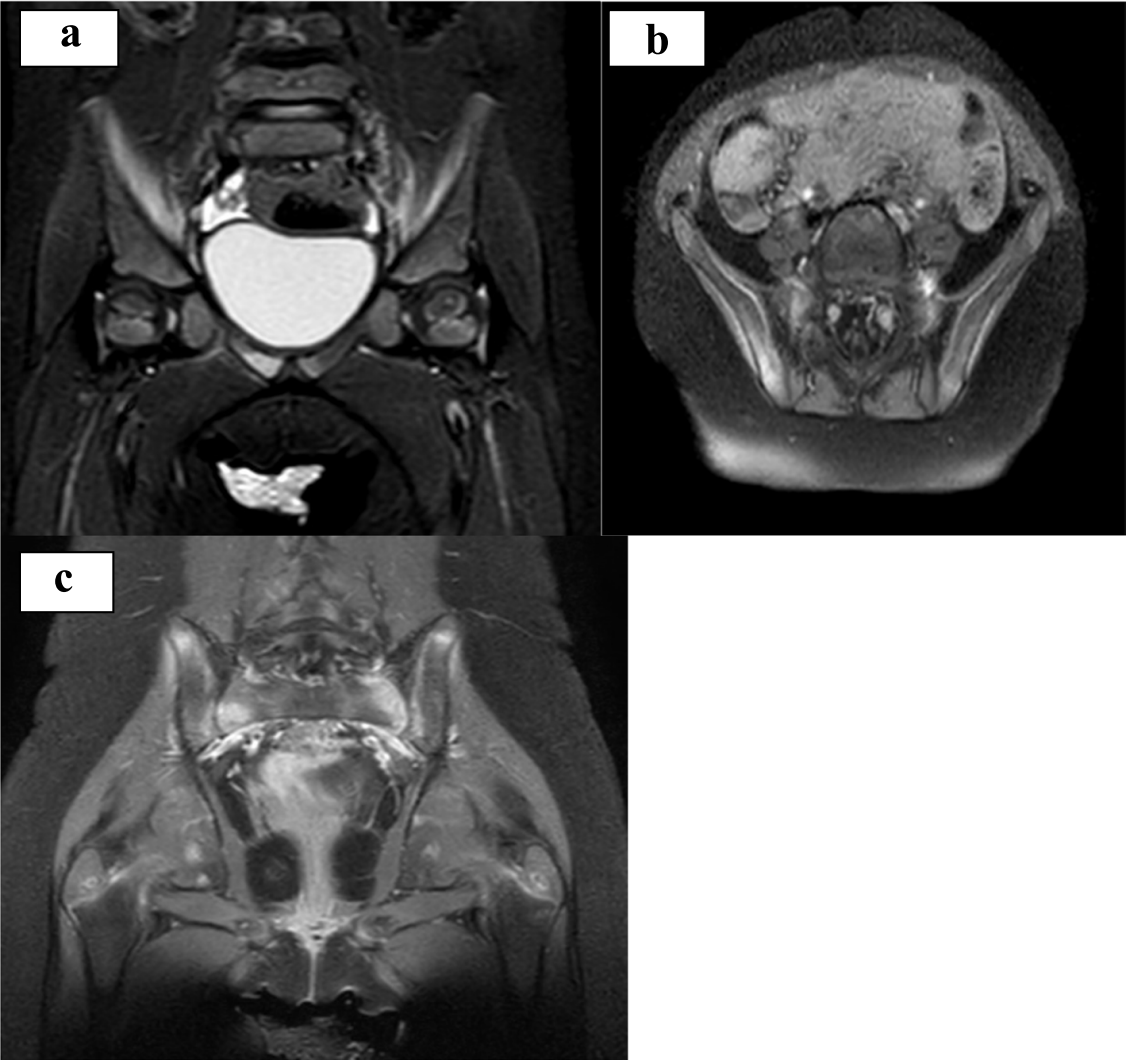
Pelvis MRI at disease onset. **a** The T2w sequence documents some areas of altered signal referable to spongious edema, some of them with symmetrical distribution, blurred, involving the limiting joints of the sacro-iliac synchondroses and more cranially the iliac crests, others with asymmetrical distribution, in the presence of conspicuous edematous alteration of the pre-symphyseal side of the right ileo- and ischio-pubic branches that on the opposite side seems only veiledly hinted at. **b**, **c** Changes are accompanied by discrete contrastographic impregnation in the CE-FS T1w sequence, particularly that located at the pubic site on the right, where concomitant periosteal reaction is seen, with traces of initial edema of the adjacent adductor muscle bundles

**Fig. 2:**
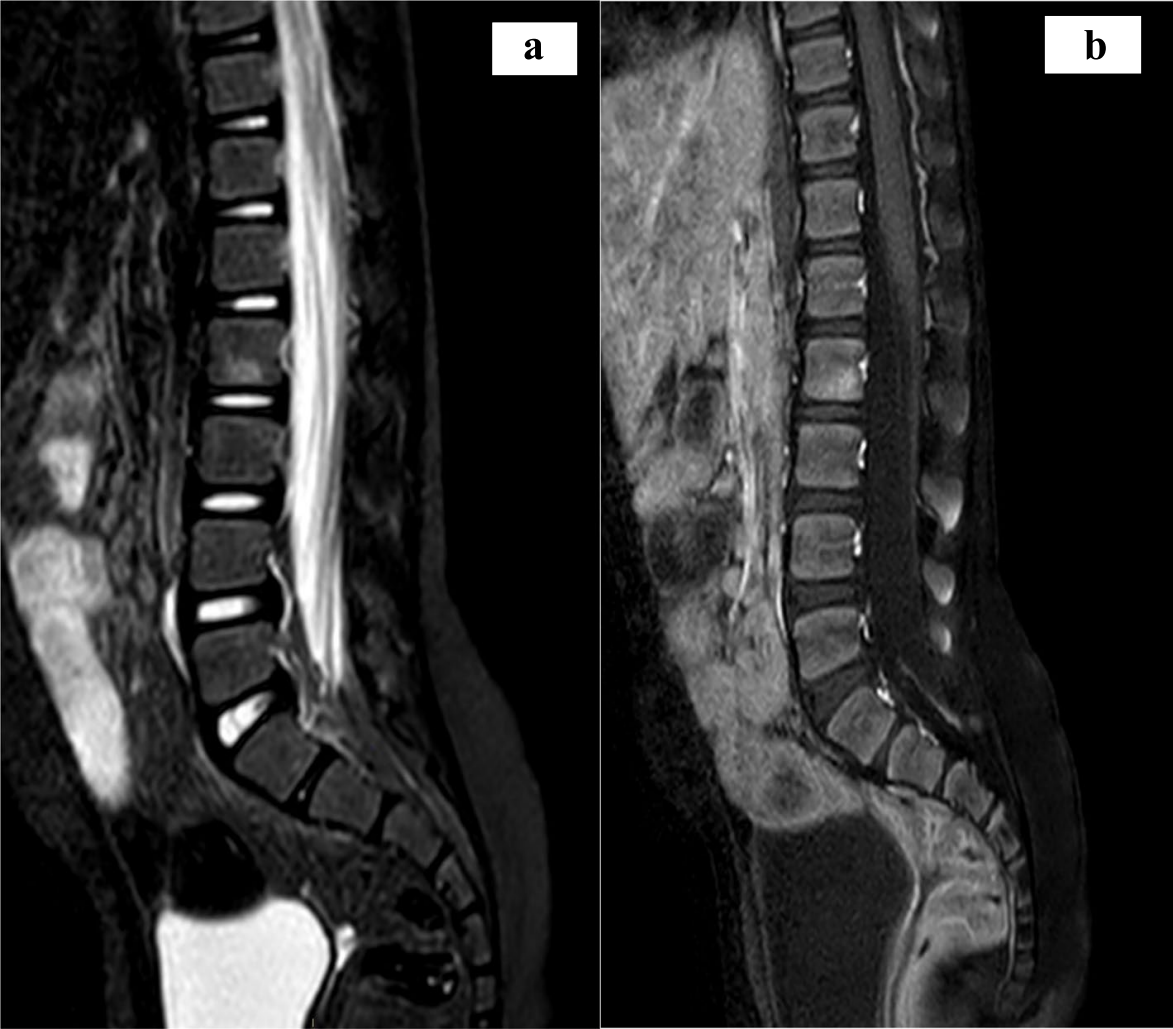
Spine MR at disease onset. At the level of the lumbar vertebrae, symmetrical alterations with similar pre- (**a** STIR) and post-contrastographic (**b** T1w CE-FS) signal are reported at the level of the transverse processes of L5 and, more nuanced, at the antero-superior edge of L1 in the right paramedian location and at the lower portion of the soma of L2

Upon the suspicion of CNO, a WBMR was performed which detected multifocal and often symmetric bone alterations involving all the skeleton but the skull. In particular, the MR showed scattered areas of bone marrow edema of the hip involving the sacroiliac joints, the iliac crest and the right ileo-ischium-pubic branch. A concomitant periostal reaction of the iliac crests and of the right ileo-ischium-pubic branch was associated to swelling of adductors and iliac muscle fascia. Similar images were found in transverse process of L1, L2 and L5, in L1 and L2 soma, although without vertebral body compression or fractures. Several other skeletal segments were interested: sternum, clavicles, coracoid process and scapular spines, all costal cartilages, proximal humerus metaphysis, ulna and radio distal metaphysis, proximal and distal metaphysis of tibia, fibula and femur. The exam did not find out joint effusion, nor lithic bone alterations (Figs. 1, 2).

In order to rule out osteomyelitis and malignancies, and to better define the bone histology, we performed bone marrow aspiration (negative for atypical cellular infiltrates), together with bone biopsy (iliac crest and a tibial lesion): both showed an overlapping and non-specific pattern, including bone trabeculae remodelling, mild inflammatory infiltrate and fibrosis; bacterial cultures tested negative.

Auto-inflammatory diseases were excluded by genetic analysis (NGS sequencing) for the principal candidate genes (PSTPIP1; LPIN2, IL1RN, IL36RN, CARD14, PLCG2, NOD2, LACC1, CECR1).

In the meanwhile the immunologic evaluation showed positive Antinuclear Antibodies (ANA, 1:160) and negative Antineutrophil Cytoplasmic Antibodies (ANCAs) and Anti-Saccharomyces Cerevisiae Antibody (ASCA), while the screening for CD resulted positive, with significant presence of both Anti-Endomysial antibody (EMA) and IgA-Anti transglutaminase antibodies (IgA-ATA). The esophagogastroduodenoscopy with biopsy showed histologic evidence of villous atrophy of mild degree (Marsh stage 3), confirming the diagnosis of CD.

Based on the radiological findings and the clinical features, the girl was diagnosed affected by CRMO and celiac disease and discharged with the prescription of gluten free diet and prosecution of the anti-inflammatory therapy *per os* (ibuprofen, 30 mg/kg/day in 3 doses) to control leg pain.

Already at the first outpatient visit, just eight days after the start of the gluten free diet, the girl did not complain of pain, so anti-inflammatory therapy was rapidly tapered and discontinued within one month. The microscopic haematuria disappeared and never appeared again. The ESR value returned to the normal value in a few weeks.

**Fig. 3:**
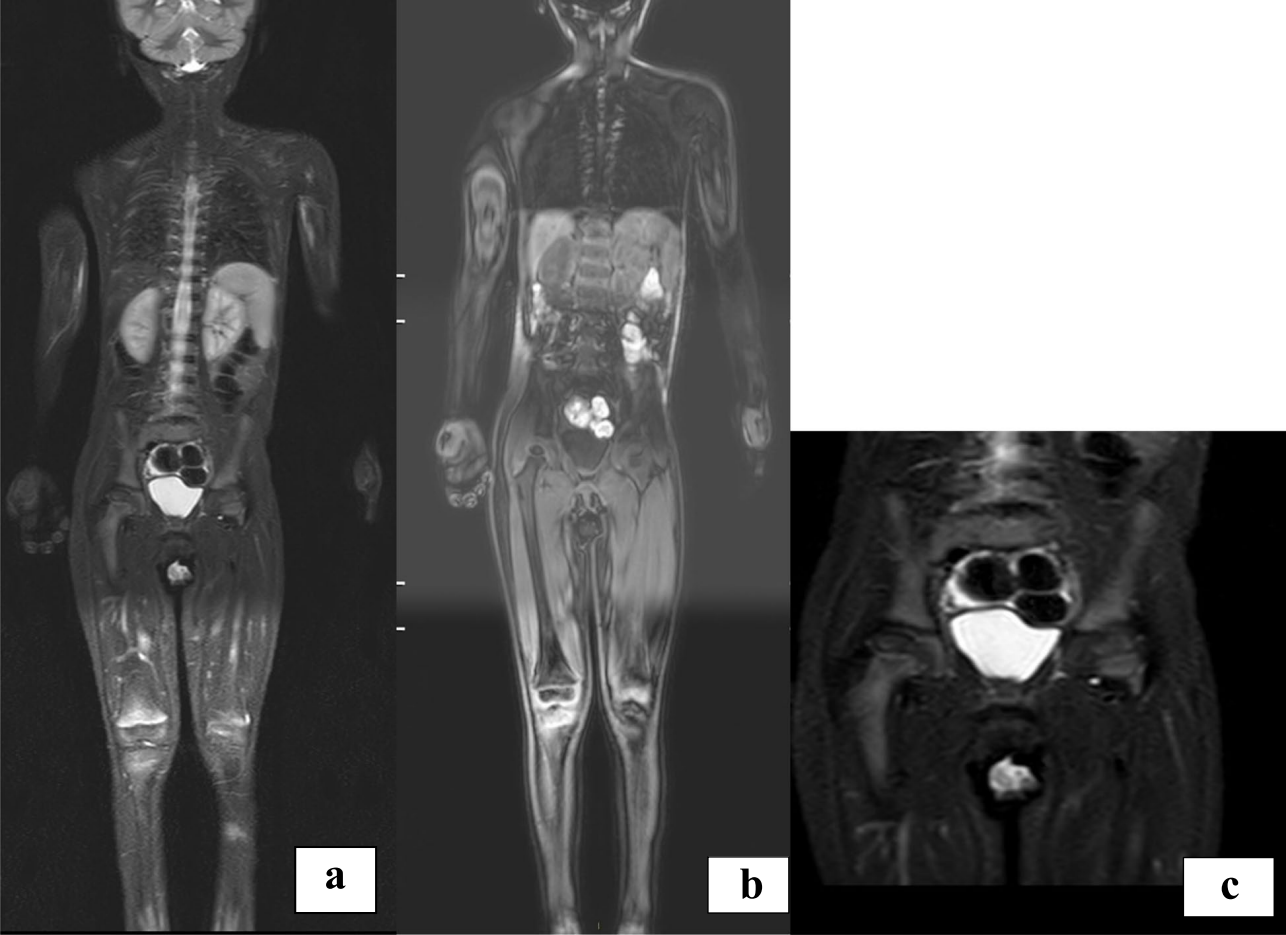
**a**, **b**, **c** WBMR four months after the gluten-free diet start. Almost complete resolution of the alterations described at the previous MRI examination. In particular, alterations previously located at the sacral flaps, iliac side of the synchondroses, iliac crests, right pubic spine, chondrocostal and sternal joints, distal clavicles and proximal humeri, distal metaphyses of radius and ulna, and acetabular roofs are no longer recognizable. Clear reduction of alterations at distal femoral and proximal tibial metaphyseal regions, where only a tinge of signal hyperintensity in STIR accompanied by a veiled T1 hypointensity remains, in the absence of significant alterations in diffusion sequences; at this level, the previously reported edematous perischelectric soft tissue alterations are no longer evident

Four months after the diagnosis the girl was healthy and asymptomatic, free from any therapy; her static-weight growth had improved and the adherence to the gluten-free diet was complete. All the laboratory tests were in the normal range and inflammatory markers values had returned to normal. IgA-ATA were negative and EMA was still slightly positive. The WBMR showed complete disease healing, with disappearance of all the bone lesions previously described (Fig. 3).

Thereafter, the little girl has been periodically evaluated in an outpatient setting. Three years later she is still in good health, her growth parameters are within the normal range and her psychomotor development is improved; she has never again complained of bone pain.

## Discussion and conclusions

CNO is a rare auto-inflammatory disorder that usually affects children, resulting from an imbalance of cytokine secretion and characterized by multiple and scattered foci of sterile osteomyelitis. It causes severe bone pain and can lead to fractures and deformities [15, 16, 46].

In 6%−18% of patients CNO is associated with extra-osseous clinical manifestations, mainly involving the skin and the gut. Among all other clinical associations, the one between CNO and the autoimmune gastrointestinal diseases is certainly the most intriguing one.

From a clinical point of view, the possible coexistence of CNO and IBD in the same patient has been known since 1999 [43] and thereafter the number of cases reported in the literature has been increasing up to our days.

Two recent comprehensive reviews analyze all the cases of CNO associated with IBD previously described in carefully selected articles. As the reference literature of these papers is largely overlapping, the content of the two reviews does not differ significantly [45, 47].

Tzaneti et al. describe 40 patients (36 children) with CNO and IBD: 57.5% of them were diagnosed with Crohn’s disease, while Ulcerative Colitis accounted for 37.5% of cases and unclassified IBD for 5% only. No case of CD is reported in this paper [47].

On the other hand, Costi et al. report about 57 cases of CNO and IBD (49 children): 60% of patients met the diagnostic criteria of Crohn’s disease, 34% were affected by Ulcerative Colitis and 6% by unclassified IBD. Also in this cohort no case of CD is described [45].

In the current literature we were able to find only one abstract that reports the experience of a single monocentric Italian cohort including three cases (13%) of pediatric patients with CNO and positive screening test for CD (IgA anti-tissue transglutaminase antibodies). For the single patient with CD diagnosis confirmed by duodenal biopsy, the Authors concisely refer that clinical remission and radiological improvement occurred after the gluten-free diet, without more detailed clinical information or iconographic documentation [48].

To the best of our knowledge, ours is the first reported case of a pediatric patient with a simultaneous double diagnosis of multifocal CNO and CD, associated with mild and transient renal involvement.

The diagnostic and radiologic work up of our patient, as well as the prompt good response to the gluten-free diet, have been thoroughly described and the radiological healing of CNO has been fully documented by WBMR. Three years after the diagnosis, our patient is healthy and never complained of bone pain again; her static-weight growth and all biochemical parameters including urinalyses are within the normal range for age.

Our experience appears significant in many clinical and scientific respects.

First of all, the prevalence of CD among Italian children has significantly increased during the last 25 years and it is now one of the highest in the world (> 1.5%) [49]. Because CD is a systemic disorder, characterized by a wide variability of clinical features encompassing a great variety of extra-intestinal manifestations, often evident before or in the absence of the classic gastrointestinal symptoms, clinicians must improve their awareness about all the possible atypical clinical CD presentation, even the rarest ones.

Some of the extra-intestinal manifestations of CD have been known for a long time and are actively investigated in CD patients, namely anemia, hepatitis, peripheral neuropathy, gluten ataxia, psychiatric conditions, endocrine disorders, dermatitis herpetiformis, and recurrent aphthous stomatitis [50, 51]. Renal involvement has also been previously described [52]. On the contrary, bone involvement, extensively documented in CD adult patients [53], is actually lesser known and investigated in children. Although it has been reported that 7%–16% of CD pediatric patients show reduced body mass index at diagnosis, the current evidence does not support routine screening of bone alterations [54, 55].

Conversely, IBD patients often complaint of extra-intestinal symptoms involving skin, eye and the musculoskeletal system with osteopenia, although arthralgia and bone pain are seldom reported as presenting symptoms [56, 57].

The coexistence of gut and bone diseases in patients affected by IBD or CD offers a unique opportunity of a “in vivo” model to improve the knowledge of the role played by the intestinal microbiota in immune system modulation.

Based on recent scientific studies showing the existence of compositional differences in oral and fecal microbiota between CNO patients and healthy subjects [58, 59], an interesting hypothesis highlighting the potential role of both dysbiosis and gut barrier disruption in the pathology of CNO has been postulated [46]. Beside that, the crucial impact of intestinal microbiota abnormalities in IBD pathogenesis has been known for a long time [60, 61].

Currently, the pathogenetic pathway linking intestinal inflammation in IBD and CD to bone inflammation in CNO still remains to be fully explained, but it is unquestionable that, regardless of the organ or tissue involved, all these clinical disorders are the result of an immunologic dysregulation that can elicit either excessive immune reactivity, with pro-inflammatory cytokine network activation, or inadequate immune response. Based on this evidence, it has been postulated that, in genetically predisposed subjects, environmental agents can act as triggers of the dysregulation process leading to inflammation in different sites, such as gut and bones [62–64].

In conclusion, as per our experience, facing a pediatric patient with newly diagnosed CD and complaining of intensive bone pain, the possibility of CNO should be taken in mind and properly investigated with WBMR.

On the other hand, due to increasing evidence of possible association between CNO and autoimmune gastro-intestinal diseases, it is adviseable to include in the diagnostic work-up of CNO, beside the inflammatory markers, also albumin, fecal calprotectin and blood assessment, CD serological screening, autoimmunity, and, in CD suspected cases, histological confirmation with gut biopsy.

This approach not only prevents missing diagnosis of both CD and IBD, but also, due to the possible therapeutic effect of the gluten-free diet on CNO, spares CD patient from unnecessarily prolonged anti-inflammatory treatments.

Moreover, the proper and timely diagnosis of asymptomatic IBD associated with CNO significantly reduces the risk of complications, although it poses significant problems related to the choice of the best therapeutic approach, which provides a good control of both conditions while reducing the potential severe adverse events. In fact, while the monoclonal antibodies anti-TNF-α may look like the most appropriate therapy for patients with IBD and CNO, the use of infliximab and adalimumab have been associated with the risk of developing psoriasis, so that more recently other types of anti-TNF (golimumab) [65], or other monoclonal antibodies inhibitors of interleukin-12/23 (ustekinumab) or IL-6 (tocilizumab) [66, 67].

In our patient the diagnosis of CNO and CD were concomitant, a scenario instead seldom reported in patients with IBD and CNO. According to the literature, CNO diagnosis precedes that of IBD in about 50% of patients. The median time interval between the two disorders presentation is usually two years, but could be as long as fifteen years [45, 47]. The possibility of such a long latency between the onset of the two diseases implies that both groups of children, those with CNO and those with gastrointestinal autoimmune diseases (IBD or CD), should be carefully monitored for a long time and throughout adulthood, in order to receive a prompt diagnosis and a proper treatment soon after the onset of suggestive symptoms or biochemical abnormalities.

## Data Availability

All the data analyzed and reported in this paper are an integral part of the patient’s medical record and are available from the corresponding author on reasonable request.

## Abbreviations

CNO: Chronic Nonbacterial Osteomyelitis
WBMR: Whole Body Magnetic Resonance
IBD: Inflammatory Bowel Disease
CD: Celiac disease
CRMO: Chronic Recurrent Multifocal Osteomyelitis
SAPHO: Synovitis, acne, pustolosis, hyperostosis and osteitis syndrome
CRP: C-reactive protein
ESR: Erythrocyte sedimentation rate
ANA: Antinuclear Antibodies
ANCAs: Antineutrophil Cytoplasmic Antibodies
ASCA: Anti-Saccharomyces Cerevisiae Antibody
EMA: Anti-Endomysial antibody
IgA-ATA: IgA-Anti transglutaminase antibodies
